# The Predictive Validity of Four Intelligence Tests for School Grades: A Small Sample Longitudinal Study

**DOI:** 10.3389/fpsyg.2017.00375

**Published:** 2017-03-13

**Authors:** Jasmin T. Gygi, Priska Hagmann-von Arx, Florine Schweizer, Alexander Grob

**Affiliations:** Department of Psychology, University of BaselBasel, Switzerland

**Keywords:** validity, scholastic achievement, IDS, RIAS, SON-R 6-40, WISC-IV

## Abstract

Intelligence is considered the strongest single predictor of scholastic achievement. However, little is known regarding the predictive validity of well-established intelligence tests for school grades. We analyzed the predictive validity of four widely used intelligence tests in German-speaking countries: The Intelligence and Development Scales (IDS), the Reynolds Intellectual Assessment Scales (RIAS), the Snijders-Oomen Nonverbal Intelligence Test (SON-R 6-40), and the Wechsler Intelligence Scale for Children (WISC-IV), which were individually administered to 103 children (*M*_age_ = 9.17 years) enrolled in regular school. School grades were collected longitudinally after 3 years (averaged school grades, mathematics, and language) and were available for 54 children (*M*_age_ = 11.77 years). All four tests significantly predicted averaged school grades. Furthermore, the IDS and the RIAS predicted both mathematics and language, while the SON-R 6-40 predicted mathematics. The WISC-IV showed no significant association with longitudinal scholastic achievement when mathematics and language were analyzed separately. The results revealed the predictive validity of currently used intelligence tests for longitudinal scholastic achievement in German-speaking countries and support their use in psychological practice, in particular for predicting averaged school grades. However, this conclusion has to be considered as preliminary due to the small sample of children observed.

## Introduction

The primary purpose of the first intelligence test (Binet and Simon, 1905) was to predict scholastic achievement in order to determine the best school setting for a child. Since the beginning of intelligence assessment, the predictive validity of intelligence test scores for scholastic achievement has been well studied. Cross-sectional and longitudinal studies indicated strong correlations, around *r* = 0.40–0.81, between the two (e.g., Sternberg et al., 2001; Deary et al., 2007; Mackintosh, 2011).

The association between intelligence and scholastic achievement seems to be stronger when using standardized achievement tests compared to school grades (Sternberg et al., 2001; Rost, 2009). Standardized achievement tests represent achievement at only one point in time, whereas school grades represent achievement over a longer period and thus may also be influenced by other constructs such as self-control and motivation (Rost, 2009). However, school grades are crucial for children to be promoted to the next higher grade level as well as for further scholastic and occupational qualifications (Roth et al., 2015).

Focusing on school grades, a recent meta-analysis (Roth et al., 2015) found an observed correlation of *r* = 0.44 and an estimated true correlation (i.e., corrected for error of measurement and range restriction) of ρ = 0.54 between intelligence and school grades. Regarding subject domains, the correlations were highest and comparable for mathematics/science (*r* = 0.42, ρ = 0.49) and languages (*r* = 0.36, ρ = 0.44). The results furthermore revealed that correlations between intelligence and school grades in elementary school (*r* = 0.40, ρ = 0.45) tended to be weaker than in middle and high school (*r* = 0.46, ρ = 0.54–0.58), because intelligence deficits in elementary school may be compensated more easily through practice than in higher-grade levels, as the learning content is easier to understand. This result is in contrast to previous research (e.g., Sternberg et al., 2001), that identified stronger correlations between intelligence and scholastic achievement in elementary school than in higher-grade levels, because of growing range restrictions.

The meta-analysis performed by Roth et al. (2015) included studies conducted in different countries. In German-speaking countries, for example, the Culture Fair Test-20-Revision (Weiss, 2006), standardized in 2003, showed associations with school grades in mathematics/science ranging from *r* = 0.26 to 0.39 and in languages of *r* = 0.23. Further, the German Cognitive Ability Test – 4-12 – Revision (KFT 4-12+R; Heller and Perleth, 2000), standardized from 1995 to 1997, showed associations with school grades in mathematics/science ranging from *r* = 0.17 to 0.60 and in languages ranging from *r* = 0.12 to 0.14. In another study, the KFT 4-12+R and the German version of the Wechsler Intelligence Scale for Children-III (Tewes et al., 1999), standardized from 1995 to 1998, predicted mathematics/science with β = 0.54 and language with β = 0.52 (Karbach et al., 2013). However, the meta-analysis did not include more recently standardized intelligence tests currently used in German-speaking countries.

Currently used intelligence tests in German-speaking countries (e.g., Hagmann-von Arx et al., 2015) include (a) the Intelligence and Development Scales (IDS; Grob et al., 2013), an intelligence test for children aged 5–10 years measuring in particular fluid intelligence; (b) the Reynolds Intellectual Assessment Scales (RIAS; Reynolds and Kamphaus, 2003; German version: Hagmann-von Arx and Grob, 2014), an intelligence test for individuals aged 3 to above 90 years that measures verbal and nonverbal intelligence, based on crystallized and fluid intelligence, respectively. A composite intelligence index can be computed from the values in verbal and nonverbal intelligence; (c) the Snijders-Oomen Nonverbal Intelligence Test Revised 6-40 (SON-R 6-40; Tellegen et al., 2012), a nonverbal intelligence test measuring fluid intelligence in individuals aged 6–40 years; and (d) the Wechsler Intelligence Scales for Children, Fourth Edition (WISC-IV; Wechsler, 2003; German version: Petermann and Petermann, 2011), an intelligence test used worldwide to measure general intelligence (Full-Scale IQ or FSIQ). Additionally, the WISC-IV provides four index scores: verbal comprehension reflecting the understanding of verbal concepts; perceptual reasoning measuring nonverbal perception and manipulation; working memory assessing attention and working memory; and processing speed reflecting visuospatial speed of processing.

Nevertheless, little is known regarding the predictive validity of these intelligence tests for school grades. Especially for German-speaking countries and for studies independent of the standardization samples, there is a lack of literature analyzing predictive validity of these tests. For the IDS, Gut et al. (2013) analyzed the predictive validity of general intelligence in children aged 5–7 years from the standardization sample for concurrent (*n* = 402) and longitudinal (*n* = 221) scholastic achievement. Concurrent scholastic achievement was operationalized through parents' and teachers' ratings in mathematics, science, and language (German), which were averaged across subjects. Longitudinal scholastic achievement was based on averaged school grades in these subjects 3 years later. Results revealed medium to large effect sizes (β = 0.30–0.56) for the cross-sectional and a small effect size (β = 0.21) for the longitudinal association. These results replicate findings of a prior study conducted by Gut et al. (2012) showing that in an extended sample of 263 children aged 5–10 years, IDS general intelligence predicted school grades in mathematics, science, and language (German) 3 years later with medium effect sizes (β = 0.28–0.34). Both studies indicate small to moderate concurrent and predictive validity of the IDS general intelligence for averaged school grades.

For the German version of the RIAS, we found no studies on the predictive validity of intelligence indices on school grades. However, the Technical Manual of the English Version of the RIAS (Reynolds and Kamphaus, 2003) reports a cross-sectional validation study conducted with 78 children aged 3–16 years. Results revealed strong correlations between the composite intelligence index and a standardized achievement test in mathematics (*r* = 0.67) and language (*r* = 0.64), indicating strong concurrent validity of the RIAS composite intelligence index for standardized achievement tests.

For the SON-R 6-40, the Technical Manual of the German version (Tellegen et al., 2012) reports moderate to strong correlations between the test scores and concurrent school grades in mathematics (*r* = 0.58) and language (*r* = 0.49) for 182 elementary school children aged 6–11 years. These results indicate that nonverbal intelligence measured using the SON-R 6-40 shows moderate to strong concurrent validity for school grades.

For the German version of the WISC-IV there are, to our knowledge, no available studies on the predictive validity for school grades. For the English version of the WISC-IV, Glutting et al. (2006) studied the concurrent validity of general intelligence and its specific indices on a standardized academic achievement test in mathematics and reading with a sample of 498 individuals aged 6–16 years from the WISC-IV standardization sample. Results showed large effect sizes for the WISC-IV FSIQ (60% variance explained) but only small effect sizes for the specific indices (0–2% additional variance explained). The FSIQ predicted concurrent mathematics and reading equally well. Thus, results indicate that in particular the WISC-IV FSIQ is correlated with concurrent standardized academic achievement tests.

In sum, Roth et al.'s (2015) meta-analysis revealed that intelligence test scores correlate moderately to strongly with school grades in mathematics and language. However, there is only scarce evidence regarding the longitudinal prediction of school grades with currently used intelligence tests in German-speaking countries.

In the current study we analyzed the predictive power of the German versions of the IDS, the RIAS, the SON-R 6-40, and the WISC-IV for longitudinal school grades. We analyzed the general intelligence indices only, as the Technical Manuals (e.g., Reynolds and Kamphaus, 2003) as well as previous research (Glutting et al., 2006) do not recommend the use of specific indices for high-stakes decisions because of lowered reliability and validity compared to the general intelligence indices. On the basis of previous research (e.g., Glutting et al., 2006; Deary et al., 2007; Gut et al., 2012, 2013; Roth et al., 2015), we expected that the general intelligence indices would positively predict averaged school grades as well as school grades in mathematics and language (German) with medium to strong effect sizes.

## Materials and methods

### Participants

The sample consisted of 103 children aged 6–11 years (*M* = 9.17 years, *SD* = 0.93; 52% females, 48% males) enrolled in regular schools. All children took part in an intelligence assessment. Three years later, parents of 54 children aged 10–13 years (*M* = 11.77 years, *SD* = 0.79; 52% females, 48% males) provided information about their children's school grades in mathematics and language. Regarding parental education, 74% of the parents had a non-tertiary education and 26% had a tertiary education. This distribution indicates that parent's educational attainment in the present study is comparable with the general Swiss population (Swiss Federal Statistical Office, 2016). Post hoc power analysis using G^*^Power (Faul et al., 2007) indicated that with a chance of 80% and a 0.05 alpha level, the current study was sufficiently powered to detect medium effect sizes (*r* = 0.30; Cohen, 1988). The 54 children who participated in both study waves showed significantly higher intelligence scores in the RIAS composite intelligence index (*M* = 103.24, *SD* = 8.29) as well as in the WISC-IV FSIQ (*M* = 107.39, *SD* = 10.58) than the 49 children who did not participate in the second study wave (RIAS: *M* = 98.98, *SD* = 9.19, *F* = 0.82, *p* < 0.01; WISC-IV: *M* = 102.12, *SD* = 12.78, *F* = 1.57, *p* < 0.05). No differences were found for the IDS and the SON-R 6-40.

### Measures

#### Intelligence

To assess intelligence (*M* = 100, *SD* = 15), the IDS, RIAS, SON-R 6-40, and WISC-IV were administered. The IDS assesses general intelligence and five developmental domains (psychomotor skills, social-emotional competences, mathematics, language, and achievement motivation) in children aged 5–10 years. For the current study, only general intelligence was analyzed. IDS general intelligence consists of seven subtests (i.e., visual perception, selective attention, phonological memory, visual-spatial memory, auditory memory, abstract reasoning, figural reasoning), which measure mainly fluid intelligence. The administration of IDS general intelligence takes about 45 min. The IDS was standardized from 2007 to 2008 in Austria, Germany, and Switzerland. Reliability for general intelligence is high with Cronbach's α = 0.92.

The RIAS is an intelligence test for individuals aged 3 to above 90 years. It comprises four intelligence subtests (i.e., guess what, verbal reasoning, odd-item out, what's missing), which together constitute the composite intelligence index, CIX. The CIX can also be divided into two indices, represented by two of the four above mentioned subtests each: the Verbal Intelligence Index, VIX, representing crystallized intelligence, and the Nonverbal Intelligence Index, NIX, representing fluid intelligence. Two additional subtests can be administered measuring verbal and nonverbal memory resulting in a Composite Memory Index. The memory subtests are not entered in the CIX. The assessment of the RIAS CIX takes about 20–25 min. The German version of the RIAS was standardized from 2011 to 2012 in Germany and Switzerland. Reliability for the RIAS is high with Cronbach's α = 0.95 for the CIX and α = 0.93–0.94 for the VIX and NIX.

The SON-R 6-40 assesses nonverbal intelligence for individuals aged 6–40 years. It comprises four subtests (i.e., analogies, categories, mosaics, patterns) that primarily measure fluid intelligence. The administration of the SON-R 6-40 takes about 45–60 min. The German version of the SON-R 6-40 was standardized from 2009 to 2011 in Germany and the Netherlands. Reliability for the SON-R 6-40 is high with Cronbach's α = 0.95.

The WISC-IV is an intelligence test measuring general intelligence for children aged 6–16 years. It includes 10 core subtests (i.e., similarities, vocabulary, comprehension, block design, picture concepts, matrix reasoning, digit span, letter-number sequencing, symbol search, coding) that constitute the FSIQ and four specific indices: the Verbal Comprehension Index, Perceptual Reasoning Index, Working Memory Index, and Processing Speed Index. The administration of the WISC-IV core subtests takes about 60 min. The German version of the WISC-IV was standardized from 2005 to 2006 in Austria, Germany, and Switzerland. Reliability for the WISC-IV is high with *r* = 0.97 for the FSIQ and *r* = 0.87–94 for the specific intelligence indices.

#### School grades

Three years after intelligence assessment, parents were asked to report on their child's school grades in mathematics and language (1 = poorest grade, 6 = best grade; grades 4–6 represent the passing range) based on the school records of the latest term (i.e., overall grades). In Switzerland, passing grades in both mathematics and language are crucial for a child to be promoted to the next higher grade level (Swiss Media Institute for Education and Culture, 2016). Thus, in line with previous research (e.g., Gut et al., 2013) school grades were additionally averaged across subjects to obtain a composite estimate of scholastic achievement.

### Procedure

This study was carried out in accordance with the recommendations of the Ethics Committee of Basel, Switzerland and with the Declaration of Helsinki. Parents gave written informed consent prior to participation in the study, and assent was obtained from the children. Children were recruited from elementary schools in the German-speaking part of Switzerland in 2011. Trained study personnel administered the tests at school on regular school days. Each child was individually administered the four intelligence tests (IDS, RIAS, SON-R 6-40, WISC-IV) in counterbalanced order. Three appointments were required, each about 2 h, including breaks (one test session for the IDS, one test session for the WISC-IV, and one test session for the RIAS and SON-R 6-40). The sample sizes for each intelligence test vary somewhat, as a few children could attend only two testing appointments (*n*_IDS_ = 103, *n*_RIAS_ = 102, *n*_SON-*R*6-40_ = 101, *n*_WISC−IV_ = 103). After the study was completed, the parents received a written report on their child's performance in each intelligence test. Three years later, parents were contacted again and asked to provide information about their child's school grades. Two families had moved and could not be reached; in all, 54 parents returned the requested information (resulting in a response rate of 53%).

### Data analyses

All analyses were conducted using SPSS 23.0. Because of the small sample size and because some of the variables showed deviations from normality (see skewness and kurtosis in Table 1), we used bootstrap procedures (Efron, 1979; Chernick, 2008). Bias-corrected 95% confidence intervals (BC 95%-CI) were computed based on 5,000 random samples. A result was considered to be significant when the confidence interval did not include zero.

**Table 1:** **Descriptive statistics for control variables, the intelligence scores of the IDS, RIAS, SON-R 6-40, WISC-IV, and school grades in study waves 1 and 2**.

**Variable**	**Study wave 1 (*****N*** = **103)**					**Study wave 2 (*****N*** = **54)**				
	**Mean (*SD*)**	***N* (%)**	**Range**	**Skewness**	**Kurtosis**	**Mean (*SD*)**	***N* (%)**	**Range**	**Skewness**	**Kurtosis**
Sex										
Female		54 (52)					28 (52)			
Male		49 (48)					26 (48)			
Age at Study wave 1 (years)	9.18 (0.93)		6.71–11.18	−0.19	0.26	9.04 (0.96)		6.71–11.18	−0.08	−0.14
Age at Study wave 2 (years)						11.77 (0.80)		10.25–13.60	0.43	−0.51
Parental education										
Non-tertiary education		74 (72)					40 (74)			
Tertiary education		29 (28)					14 (26)			
IDS	103.54 (10.64)		78–134	0.15	0.41	105.19 (10.74)		79–134	0.45	0.70
RIAS	101.45 (8.99)		81–128	0.29	0.39	103.65 (8.23)		87–128	0.84	0.67
SON-R 6-40	103.27 (11.32)		79–139	0.38	0.50	103.67 (11.55)		82–139	0.62	0.61
WISC-IV	104.88 (11.91)		73–133	0.21	0.07	107.39 (10.58)		84–133	0.27	0.28
Averaged school grade						5.16 (0.42)		4–6	−0.39	−0.20
Mathematics school grade						5.15 (0.48)		4–6	−0.25	−0.71
Language school grade						5.16 (0.43)		4–6	−0.31	−0.08

*IDS, Intelligence and Development Scales; RIAS, Reynolds Intellectual Assessment Scales; SON-R 6-40, Snijders-Oomen Nonverbal Intelligence Test Revised; WISC-IV, Wechsler Intelligence Scale for Children—Fourth Edition*.

To analyze the predictive validity of each intelligence test, separate regression analyses for each predictor (i.e., general intelligence indices) and outcome variable (i.e., child's school grades) were conducted. All variables entered into the regression analyses were z-standardized. Few children were identified as outliers with scores more than two standard deviations from the mean (*n*_IDS_ = 2, *n*_RIAS_ = 1, *n*_SON-*R*6-40_ = 2, *n*_WISC−IV_ = 2), and for this reason these scores were truncated to z ± 2. In the following analyses, we controlled for variables that showed correlations with the outcome variables to some extent (i.e., sex, age; see Table 2).

**Table 2:** **Correlations among control variables, the intelligence scores of the IDS, RIAS, SON-R 6-40, WISC-IV, and school grades**.

**Variable**		***N***	**1**	**2**	**3**	**4**	**5**	**6**	**7**	**8**	**9**	**10**
1	Sex (0 = males, 1 = females)	54	1									
2	Age at Study wave 1	54	−0.09	1								
3	Age at Study wave 2	54	−0.09	0.79^***^	1							
4	Parental education (0 = non-tertiary, 1 = tertiary)	54	−0.02	−0.14	−0.06	1						
5	IDS	54	0.16	−0.34^*^	−0.24	0.13	1					
6	RIAS	54	−0.03	−0.31^*^	−0.13	0.15	0.75^***^	1				
7	SON-R 6-40	52	0.05	−0.16	−0.18	0.11	0.80^***^	0.63^***^	1			
8	WISC-IV	54	0.09	−0.27^*^	−0.07	−0.02	0.77^***^	0.67^***^	0.63^***^	1		
9	Averaged school grade	54	0.24	−0.30^*^	−0.37^**^	0.10	0.47^**^	0.34^**^	0.36^*^	0.30^*^	1	
10	Mathematics school grade	54	0.14	−0.25	−0.28^*^	0.13	0.43^**^	0.26	0.32^*^	0.25	0.93^***^	1
11	Language school grade	53	0.32^*^	−0.30^*^	−0.41^**^	0.06	0.44^**^	0.36^**^	0.32^*^	0.30^*^	0.91^***^	0.68^***^

*Correlations were calculated for individuals who participated at Study wave 2. IDS, Intelligence and Development Scales; RIAS, Reynolds Intellectual Assessment Scales; SON-R 6-40, Snijders-Oomen Nonverbal Intelligence Test Revised; WISC-IV, Wechsler Intelligence Scale for Children—Fourth Edition*.

* *p < 0.05*.

** *p < 0.01*.

*** *p < 0.001*.

## Results

Table 1 gives an overview of the descriptive statistics of the current sample. The mean scores of the intelligence tests were somewhat higher than in the standardization samples (*M* = 100), and the standard deviations were somewhat lower than in the standardization samples (*SD* = 15). The range of school grades is narrow (4–6) and reflects grades in the passing range. Correlations among all variables are displayed in Table 2. The general intelligence indices of all four tests correlated highly with each other (*r* = 0.63–0.80, *p* < 0.001).

Table 3 presents the results of the prediction of longitudinal school grades by intelligence (model a: IDS, model b: RIAS, model c: SON-R 6-40, model d: WISC-IV) while controlling for children's sex and age. When school grades were averaged across subjects, the general intelligence indices of all four intelligence tests predicted scholastic achievement. Estimates ranged from Estimate = 0.126 (SE = 0.054, BC 95%-CI = [0.019, 0.225]) for SON-R 6-40 to Estimate = 0.175 (SE = 0.059, BC 95%-CI = [0.056, 0.293]) for IDS.

**Table 3:** **Regression analyses for the intelligence scores of the IDS, RIAS, SON-R 6-40, and WISC-IV predicting longitudinal school grades**.

**Variables**	**Averaged school grade**				**Mathematics**				**Language**			
	**Estimate**	**SE**	**BC 95%−CI**	***R*^2^**	**Estimate**	**SE**	**BC 95%−CI**	***R*^2^**	**Estimate**	**SE**	**BC 95%−CI**	***R*^2^**
Model a: IDS												
Step 1				0.180^*^				0.092				0.251^*^
Sex (0 = males, 1 = females)	0.086	0.052	[−0.010, 0.176]		0.112	0.122	[−0.118, 0.324]		0.264	0.110	[0.055, 0.473]	
Age	−0.165	0.060	[−0.281, −0.019]		−0.281	0.140	[−0.540, 0.063]		−0.396	0.115	[−0.622, −0.137]	
Step 2^a^				0.318^*^				0.220^**^				0.341^**^
IDS	0.175	0.059	[0.056, 0.293]		0.376	0.143	[0.085, 0.677]		0.318	0.130	[0.066, 0.573]	
Model b: RIAS												
Step 1				0.180^*^				0.092				0.251^*^
Sex (0 = males, 1 = females)	0.086	0.052	[−0.007, 0.177]		0.112	0.120	[−0.127, 0.338]		0.264	0.112	[0.059, 0.476]	
Age	−0.165	0.060	[−0.266, −0.043]		−0.281	0.139	[−0.518, 0.031]		−0.396	0.117	[−0.621, −0.150]	
Step 2^a^				0.275^*^				0.148				0.347^**^
RIAS	0.165	0.066	[0.031, 0.298]		0.283	0.142	[0.023, 0.544]		0.376	0.153	[0.092, 0.699]	
Model c: SON−R 6−40												
Step 1				0.179^*^				0.093				0.251^*^
Sex (0 = males, 1 = females)	0.069	0.053	[−0.030, 0.164]		0.086	0.126	[−0.151, 0.310]		0.220	0.112	[0.013, 0.418]	
Age	−0.170	0.060	[−0.284, −0.031]		−0.293	0.141	[−0.554, 0.072]		−0.406	0.114	[−0.625, −0.165]	
Step 2^a^				0.262^*^				0.168^*^				0.301^*^
SON−R 6−40	0.126	0.054	[0.019, 0.225]		0.275	0.137	[0.007, 0.519]		0.217	0.116	[−0.020, 0.443]	
Model d: WISC−IV												
Step 1				0.180^*^				0.092				0.251^*^
Sex (0 = males, 1 = females)	0.086	0.051	[−0.013, 0.186]		0.112	0.120	[−0.114, 0.328]		0.264	0.114	[0.048, 0.476]	
Age	−0.165	0.060	[−0.270, −0.029]		−0.281	0.140	[−0.531, 0.067]		−0.396	0.115	[−0.615, −0.146]	
Step 2^a^				0.247^*^				0.140				0.304^**^
WISC−IV	0.128	0.063	[0.001, 0.266]		0.242	0.157	[−0.088, 0.590]		0.260	0.137	[−0.001, 0.553]	

*IDS, Intelligence and Development Scales; RIAS, Reynolds Intellectual Assessment Scales; SON-R 6-40, Snijders-Oomen Nonverbal Intelligence Test Revised; WISC-IV, Wechsler Intelligence Scale for Children—Fourth Edition. BC 95%-CI, bias-corrected 95% bootstrap confidence intervals*.

a *Controlled for variables in step 1*.

* *p < 0.01*,

** *p < 0.001*.

Regarding mathematics, results show that IDS (Estimate = 0.376, SE = 0.143, BC 95%-CI = [0.085, 0.677]), RIAS (Estimate = 0.283, SE = 0.142, BC 95%-CI = [0.023, 0.544]), and SON-R 6-40 (Estimate = 0.275, SE = 0.137, BC 95%-CI = [0.007, 0.519]) were significant predictors, whereas WISC-IV was not significantly related to school grades in mathematics. Regarding language, IDS (Estimate = 0.318, SE = 0.130, BC 95%-CI = [0.066, 0.573]) and RIAS (Estimate = 0.376, SE = 0.153, BC 95%-CI = [0.092, 0.699]) significantly predicted school grades. The general intelligence indices of SON-R 6-40 and WISC-IV did not predict school grades in language.

## Discussion

Our main goal was to assess the longitudinal predictive validity of four intelligence tests currently used in German-speaking countries for children's school grades in mathematics, language, and averaged across subjects.

The general intelligence indices of all four intelligence tests showed significant predictive validity for averaged school grades three years after intelligence assessment, which is in line with previous studies, showing that intelligence is a positive predictor of scholastic achievement (Deary et al., 2007; Gut et al., 2012, 2013; Roth et al., 2015). Therefore, our results support the use of the general intelligence indices of the IDS, RIAS, SON-R 6-40, and WISC-IV in order to make predictions of a child's averaged school grades.

Regarding the prediction of mathematics, IDS, RIAS, and SON-R 6-40 were significantly associated with school grades. IDS general intelligence includes four out of seven subtests that tax phonological and visual-spatial working memory (i.e., selective attention, phonological memory, visual-spatial memory, auditory memory). Previous research revealed that both aspects of working memory are associated with mathematics (e.g., Dehn, 2008; Raghubar et al., 2010). The RIAS includes two out of four subtests taxing visual-spatial abilities, while the SON-R 6-40 assesses intelligence through subtests measuring primarily visual-spatial abilities. Previous literature found visual-spatial abilities to be moderately associated with mathematics (e.g., Wai et al., 2009; Verdine et al., 2014). The WISC-IV did not significantly predict school grades in mathematics, although the small effect sizes were positive; however, this contradicts the results of previous studies that found general intelligence to be a moderate to strong predictor of school grades in mathematics (Roth et al., 2015). The WISC-IV includes perceptual reasoning and phonological working memory as two out of four specific indices, and thus measure visual-spatial abilities and working memory to a lesser extent. This might have weakened the relation between these general intelligence indices and school grades in mathematics, as visual-spatial abilities (e.g., Verdine et al., 2014; Wai et al., 2009) and working memory (Dehn, 2008; Raghubar et al., 2010) were found to be predictors of mathematics. Thus, in the IDS, phonological and visual-spatial working memory capacity, and in the RIAS and SON-R 6-40, visual-spatial abilities are considered more important parts of intelligence compared to the other intelligence tests. Therefore, it might be plausible that in particular IDS, RIAS, and SON-R 6-40 were significantly associated with school grades in mathematics.

Regarding the prediction of language, the general intelligence indices of the IDS and RIAS were significantly associated with school grades in language. The association between the IDS and language is in line with studies revealing a moderate to strong relationship between working memory and language (e.g., Dehn, 2008). The association between the RIAS and language might be explained through the high requirements of verbal abilities and verbal reasoning in two out of four RIAS subtests. The other general intelligence indices showed no significant associations with language, although the small effect sizes were positive; however, this result contradicts the findings of previous studies that found general intelligence to be a moderate to strong predictor of school grades in language (Roth et al., 2015). In contrast to the RIAS, the SON-R 6-40 focuses only on nonverbal intelligence. Furthermore and in contrast to the IDS, the SON-R 6-40 does not include subtests taxing working memory, which is considered as being associated with language (e.g., Dehn, 2008). The WISC-IV includes a specific index for verbal comprehension and working memory. However, it might be possible that these two out of four specific indices were not sufficient to significantly explain variance in school grades in language in the present study. Thus, the different subtests underlying the general intelligence indices of the IDS, RIAS, SON-R 6-40, and WISC-IV may be responsible for the different associations with school grades in mathematics and language. However, future studies with larger sample sizes have to be conducted to analyze this assumption.

It is notable that the effect sizes in the present study were in the small to moderate range and thus somewhat lower than we expected based on the meta-analytical results by Roth et al. (2015). However, in a single study, the expected effects may be smaller, as seen, for example, in Gut et al. (2012, 2013), for several reasons. In the present study, for instance, the analyzed sample showed slightly higher general intelligence scores than the population and had a narrow range in school grades, which were all in the passing range. This might have led to range restrictions, which may have weakened the correlations between intelligence and school grades in the present study (Sternberg et al., 2001; Roth et al., 2015). Also, the present study analyzed the predictive validity of intelligence tests on school grades for elementary school children. According to Roth et al. (2015), lower intelligence scores in elementary school might be better compensated with practice than in higher-grade levels, which may also have led to smaller effect sizes in the present study.

Regarding the control variables, in the present study sex was significantly related to school grades such that girls achieved higher scores in language than boys. This result is in accordance with a recent meta-analyses showing that females have advantages in school marks which are largest for languages courses (Voyer and Voyer, 2014). Furthermore, age was negatively related to school grades such that older children achieved lower school grades than younger children. A possible explanation for this relation may be that age is related to pubertal status and that a more advanced physical pubertal status is related to lower achievement motivation (i.e., academic self-efficacy and valuing of school) that in turn is related to lower achievement (Martin and Steinbeck, 2017).

In sum, our results indicate that the general intelligence indices of the German versions of the IDS, RIAS, SON-R 6-40, and the WISC-IV significantly predicted averaged school grades over three years. Furthermore, the IDS and RIAS were positively associated with longitudinal mathematics and language school grades, while SON-R 6-40 was a predictor of mathematics school grades. Thus, our results provide evidence for predictive validity of these intelligence tests (Neukrug and Fawcett, 2015).

The current study has strengths and limitations. It is a strength that we analyzed four intelligence tests currently used in German-speaking countries, as there is a paucity of information regarding their predictive validity for school grades. Furthermore, we assessed intelligence three years prior to school grades being inquired and could therefore analyze their predictive validity longitudinally. This is especially relevant when practitioners use intelligence scores in order to predict future scholastic achievement. Finally, we measured a child's scholastic achievement in mathematics and language using school grades, which reflect a child's performance and effort over an extended period of time and which are crucial for further scholastic and occupational qualifications (Roth et al., 2015). However, the association between intelligence and scholastic achievement may vary with different operationalization of scholastic achievement. In order to avoid potential errors in parental reports, future studies analyzing currently used intelligence tests in German-speaking countries might also consider achievement tests, which measure specific scholastic abilities at a specific point in time (Rost, 2009), as well as official school records obtained directly from schools. Moreover, as school grades are considered as indicators of achievement over a longer time period, they may be influenced not only by intelligence but also by other constructs (Rost, 2009). Therefore, future studies might also consider noncognitive factors that additionally predict scholastic achievement, such as school engagement (Reyes et al., 2012), motivation (Steinmayr and Spinath, 2009), self-control (Duckworth et al., 2012), personality (Poropat, 2009), and social-emotional competencies (Gut et al., 2012).

Furthermore, the current study had a high drop-out rate (although comparable to that of the studies conducted by Gut et al., 2012, 2013) for the longitudinal information on the child's school grades. This led to a small sample size at Study wave 2. The statistical power of the present study was sufficient to detect expected moderate associations, but there was not enough statistical power to detect weak associations between intelligence and school grades, as discussed above. Furthermore, the present study examined typically developing children enrolled in regular school with slightly higher intelligence. Thus, the conclusions based on the current study cannot be generalized to children with special needs or with different intelligence levels. To examine the predictive validity of the present intelligence tests, future studies are required with larger sample sizes and including children with different levels of intelligence (e.g., children with intellectual disabilities) or special needs as seen in the studies of Canivez et al. (2014), Mayes and Calhoun (2007), as well as Nelson and Canivez (2012). Because of these limitations, conclusions from the current study have to be considered as preliminary.

In conclusion, general intelligence measured with the German version of the IDS, RIAS, SON-R 6-40, and WISC-IV was a positive predictor of averaged school grades in the current longitudinal study. These results support the use of the four intelligence tests for issues raised in psychological practice and reveal their predictive validity on longitudinal scholastic achievement in typically developing school-aged children with slightly higher intelligence. Furthermore, the IDS and RIAS could predict both school grades in mathematics and language, while the SON-R 6-40 could predict school grades in mathematics. These results suggest that school grades in mathematics and language can be predicted by intelligence tests depending on their composition of subtests (e.g., working memory, verbal abilities, visual-spatial abilities). Thus, in psychological practice, examiners have to consider the variety of subtests included in a particular intelligence test when making specific predictions of mathematics and language. More studies analyzing larger samples as well as children with different levels of intelligence or special needs are required to replicate and generalize the findings of the current study.

## Author contributions

JG and PH contributed to the study design, acquisition, analysis, and interpretation of data. Drafted and revised the manuscript, gave final approval, and agree to be accountable for all aspects of the work in ensuring that questions related to the accuracy or integrity of any part of the work are appropriately investigated and resolved. FS contributed to the study design and acquisition of data. Revised the manuscript, gave final approval, and agrees to be accountable for all aspects of the work in ensuring that questions related to the accuracy or integrity of any part of the work are appropriately investigated and resolved. AG contributed to the study design and interpretation of data. Revised the manuscript, gave final approval, and agrees to be accountable for all aspects of the work in ensuring that questions related to the accuracy or integrity of any part of the work are appropriately investigated and resolved.

### Conflict of interest statement

The authors declare that the research was conducted in the absence of any commercial or financial relationships that could be construed as a potential conflict of interest.
